# Overall Survival, Treatment Duration, and Rechallenge Outcomes With ICI Therapy for Recurrent or Metastatic HNSCC

**DOI:** 10.1001/jamanetworkopen.2024.28526

**Published:** 2024-08-19

**Authors:** Lova Sun, Roger B. Cohen, Christopher A. D’Avella, Aditi P. Singh, Jonathan D. Schoenfeld, Glenn J. Hanna

**Affiliations:** 1 Division of Hematology Oncology, Perelman School of Medicine, University of Pennsylvania, Philadelphia; 2 Dana Farber Cancer Institute, Boston, Massachusetts; 3 Brigham and Women’s Hospital, Boston, Massachusetts

## Abstract

**Importance:**

Immune checkpoint inhibition (ICI) is a frontline treatment for recurrent or metastatic head and neck squamous cell carcinoma (R/M HNSCC), but questions remain surrounding optimal duration of therapy, benefits and risks of ICI rechallenge, and efficacy in first vs subsequent lines of therapy.

**Objectives:**

To estimate survival in US patients receiving ICI-based treatment for R/M HNSCC, compare outcomes associated with treatment discontinuation vs continuation at 1 or 2 years, and assess outcomes after immunotherapy rechallenge.

**Design, Setting, and Participants:**

This retrospective, population-based cohort study included adult patients in the Flatiron Health nationwide oncology database treated with immunotherapy for R/M HNSCC from 2015 to 2023. Data cutoff was August 31, 2023; data analysis was conducted from December 2023 to February 2024.

**Exposures:**

Treatment continuation vs discontinuation at 1 and 2 years; rechallenge with ICI after at least a 60-day period off ICI therapy without intervening systemic treatment (immediate rechallenge), or with intervening systemic treatment (delayed rechallenge).

**Main Outcomes and Measures:**

Overall survival (OS) from ICI initiation was analyzed using the Kaplan-Meier method. Cox multivariable regression was used to examine associations of key variables (line of therapy, human papillomavirus [HPV] status, Eastern Cooperative Oncology Group [ECOG] performance status) with survival.

**Results:**

The cohort included 4549 patients with R/M HNSCC who received ICI-containing therapy (median [IQR] age, 66 [59-72] years; 3551 [78.1%] male; 56 [1.2%] Asian, 260 [5.7%] Black or African American, 3020 [66.4%] White, 1213 [26.7%] other or unknown race; 3226 [70.9%] ECOG performance status 0 or 1). There were 3000 patients (65.9%) who received ICI in frontline and 1207 (26.5%) in second line; 3478 patients (76.5%) received ICI monotherapy. Median (IQR) OS was 10.9 (4.1-29.1) months and was longer in patients who received ICI in frontline therapy (12.2 [4.8-32.0] vs 8.7 [3.2-22.4] months), had HPV-positive cancer (16.6 [6.5-43.9] vs 8.8 [3.5-24.0] months), and had ECOG performance status 0 or 1 (13.5 [5.2-33.9] vs 5.5 [2.0-13.7] months). There were no survival differences on adjusted analysis between patients who stopped vs those who continued ICI at 1 or 2 years. Median (IQR) OS after ICI rechallenge was 15.7 (13.7-21.9) months in the immediate rechallenge group and 9.9 (3.7-18.1) months in the delayed rechallenge group.

**Conclusions and Relevance:**

In this large cohort study of patients with R/M HNSCC receiving ICI-based therapy, survival estimates closely mirrored clinical trial results, both in frontline and later-line settings. Discontinuation of ICI in long-term responders at 1 or 2 years may be a reasonable strategy that does not appear to compromise survival. ICI rechallenge was associated with clinical benefit in a subset of patients.

## Key Points

**Question:**

How do outcomes with immunotherapy for recurrent or metastatic head and neck squamous cell carcinoma (R/M HNSCC) in US oncology practices compare with clinical trial results, and should treatment be stopped at 1 or 2 years or continued indefinitely?

**Findings:**

In this cohort study of 4549 patients with R/M HNSCC treated with immunotherapy, survival was similar to clinical trial reports, and there was no survival difference associated with stopping vs continuing immunotherapy at 1 or 2 years.

**Meaning:**

These results suggest that immunotherapy has good efficacy in R/M HNSCC across patient subgroups and lines of therapy, which may be maintained after treatment discontinuation in long-term responders.

## Teaser

This cohort study investigates survival in US patients receiving immune checkpoint inhibitor–based treatment for recurrent or metastatic head and neck squamous cell carcinoma (HNSCC), outcomes of treatment discontinuation vs continuation at 1 or 2 years, and outcomes after immunotherapy rechallenge.

## Introduction

The KEYNOTE-048 (KN-048) study^1^ established the immune checkpoint inhibitor (ICI) pembrolizumab, with or without chemotherapy, as standard frontline treatment for patients with recurrent or metastatic head and neck squamous cell carcinoma (R/M HNSCC). Given concerns regarding generalizability of clinical trial results due to strict inclusion criteria and other factors, it is crucial to evaluate ICI efficacy and outcomes in routine clinical practice. Although small retrospective analyses have shown similar survival outcomes to KN-048,^2^ key questions surrounding the optimal use of ICI in R/M HNSCC remain.

KN-048 and other seminal studies of ICI in R/M HNSCC prescribed treatment for up to 2 years.^1,3,4^ The optimal duration of ICI therapy in responding patients is nevertheless unknown, as is whether there is added utility or detriment of longer-term or indefinite ICI treatment for patients with HNSCC. The value of ICI rechallenge after stopping initial ICI for toxicity, progression, or patient and/or clinician preference remains similarly unclear, with case reports describing anecdotal responses following rechallenge.^5^

In a prior observational study of long-term ICI treatment in lung cancer, we showed that there was no survival benefit associated with indefinite duration ICI treatment compared with discontinuation at 2 years.^6^ Establishing optimal treatment duration is important to avoid overtreatment and limit the substantial physical and financial toxicity associated with long-term or indefinite ICI treatment.^7^ As randomized trials of stopping vs continuing ICI in long-term responders with R/M HNSCC are unlikely to be performed, observational data provides a valuable opportunity to investigate this and other understudied questions surrounding ICI use.

We used a nationwide US oncology database to investigate patterns and survival outcomes in a US population who (1) received ICI in any line of therapy for R/M HNSCC, (2) discontinued ICI treatment at 1 and 2 years, and (3) were rechallenged with ICI after stopping therapy for at least 2 months, or after an intervening non-ICI therapy. We aimed to examine overall survival and associated baseline factors and characterize outcomes in key subgroups who discontinued treatment in the absence of progression and who were rechallenged with ICI-based therapy.

## Methods

### Cohort and Line of Therapy Definitions

This retrospective cohort study used the Flatiron Health longitudinal database comprising deidentified patient-level structured and unstructured data, curated via technology-enabled abstraction.^8,9^ During the study period, the deidentified data originated from up to 280 US cancer clinics (approximately 800 sites of care). The study included patients treated from January 1, 2015, to August 1, 2023; data cutoff was August 31, 2023; and data analysis was conducted from December 2023 to February 2024. Our cohort included patients who received ICI-containing therapy for R/M HNSCC. We defined patients’ first line of therapy (LoT) as the first systemic therapy for R/M HNSCC started at least 60 days after radiation to the primary site; subseuqent LoTs were defined per previously established definitions for this database.^10^

This study was deemed exempt from full review and was granted waiver of informed consent by the University of Pennsylvania institutional review board because it was a retrospective database study with deidentified data. We followed the Strengthening the Reporting of Observational Studies in Epidemiology (STROBE) reporting guideline.

### Statistical Analysis

Statistical analysis was performed using Stata version 15.1 (StataCorp) from December 2023 to February 2024. A 2-sided *P* < .05 was considered statistically significant.

#### Survival Definitions and Analysis

Survival analyses were performed using the Kaplan-Meier method. Overall survival (OS) was defined as time from ICI treatment start to death, or censored at last follow-up. Time to next treatment (TTNT) was defined as time from ICI treatment start to start of next LoT. Kaplan-Meier method was used to estimate OS in the total population and in subgroups of interest (ICI LoT, HPV status, and Eastern Cooperative Oncology Group (ECOG) performance status 0-1 vs ≥2). Cox multivariable regressions were used to examine associations between covariates (age, sex, race, smoking history, primary cancer site, ECOG performance status, year of treatment, programmed death ligand 1 (PD-L1) combined positive score (CPS), socioeconomic status, academic vs community practice setting, insurance type, and line of ICI therapy) and survival. Missing data were coded as a separate category. Proportional hazards assumption was assessed using Schoenfeld residuals.

#### Discontinuation Analysis Definitions

Definitions for ICI treatment discontinuation at 1 and 2 years in the absence of death or next treatment were adapted from prior work,^6^ and shown in eFigure in Supplement 1. Discontinuation at 1 year was defined as a duration of ICI-containing LoT from 335 to 394 days, without next treatment initiation or death within 90 days, whereas continuation beyond 1 year was defined as duration of ICI-containing LoT for at least 395 days, without death within 90 days of 1 year (425 days). Similarly, discontinuation at 2 years was defined as duration of ICI-containing LoT from 700 to 759 days, without next treatment initiation or death within 90 days; whereas continuation beyond 2 years was defined as duration of ICI-containing LoT for at least 760 days, without death within 90 days of 2 years (790 days). Kaplan-Meier analysis and Cox multivariable regression was used to estimate and compare OS between patients who discontinued vs continued ICI therapy at the 1- and 2-year time points.

#### Rechallenge Cohort Definitions

To assess outcomes after ICI rechallenge, we defined 2 cohorts who stopped their initial ICI-containing LoT, and then received ICI again (either the same agent or a different agent within the same class) after an intervening ICI-free interval. Immediate rechallenge was defined as initiation of an ICI-containing regimen after at least a 60-day gap after stopping the initial ICI-containing LoT, without intervening non-ICI therapy. Delayed rechallenge was defined as a non–ICI-containing LoT between initial ICI-containing LoT and another ICI-containing LoT. For both groups, OS and TTNT from start of ICI rechallenge were estimated using Kaplan-Meier analysis and displayed using box-and-whisker plots.

## Results

### Cohort Description

Our cohort consisted of 4549 patients with R/M HNSCC who received ICI-containing therapy in any LoT; median (IQR) age was 66 (59-72) years; 3551 [78.1%] were male; 56 [1.2%] were Asian, 260 [5.7%] were Black or African American, 3020 [66.4%] were White, and 1213 [26.7%] were other or unknown race; and 3226 [70.9%] had ECOG performance status of 0 or 1 (Table). The most common subsites were HPV-positive oropharynx (1365 [30.0%]) and oral cavity (1049 [23.1%]), followed by larynx (919 [20.2%]) and HPV-negative oropharynx (824 [18.1%]). Patterns of recurrence included locoregional only (804 [18%]), distant metastasis only (2163 [48%]), or both (459 [10%]), with 1123 patients (25%) without a recorded pattern of recurrence. More than half (55%) did not have a recorded PD-L1 CPS test result; of those with known PD-L1 (n = 2029), 20% had CPS of 0, 41% had CPS between 1 and 19, and 39% had CPS of 20 or more. There were 3000 patients (65.9%) who received ICI in frontline, 1207 (26.5%) as a second-line treatment, and 342 (7.5%) in third line or later line. Patients’ first ICI-containing LoT was predominantly ICI monotherapy (3478 [76.5%]); only 1071 (23.5%) received chemoimmunotherapy as their first ICI-containing LoT.

**Table.** Baseline Characteristics^a^

Characteristic	No. (%)
Patients, No.	4549
Age, median (IQR), y	66 (59-72)
Sex	
Male	3551 (78.1)
Female	998 (21.9)
Race	
Asian	56 (1.2)
Black or African American	260 (5.7)
White	3020 (66.4)
Other/unknown race^b^	1213 (26.7)
Year of 1L treatment	
2015-2017	986 (21.7)
2018-2020	1839 (40.4)
2021-2023	1724 (37.9)
ECOG performance status	
0	1114 (24.5)
1	2112 (46.4)
2	726 (16.0)
3	156 (3.4)
4	10 (0.2)
Missing	431 (9.5)
Smoking history	3501 (77.0)
Primary site	
HPV-positive oropharynx	1365 (30.0)
HPV-negative oropharynx	824 (18.1)
Larynx	919 (20.2)
Oral cavity	1049 (23.1)
Hypopharynx	282 (6.2)
Unknown primary	110 (2.4)
PD-L1 CPS	
0	401 (8.8)
1-19	839 (18.4)
≥20	789 (17.3)
Unknown	2520 (55.4)
Line of first ICI-containing therapy	
1	3000 (65.9)
2	1207 (26.5)
≥3	342 (7.5)
First ICI-containing line of therapy	
ICI monotherapy	3478 (76.5)
ICI + chemotherapy	1071 (23.5)
Practice type	
Community	3482 (76.5)
Academic	1067 (23.5)
Geographic location	
West	480 (14.4)
Midwest	442 (13.3)
Northeast	304 (9.1)
South	2104 (63.2)
Insurance type	
Commercial	2498 (54.9)
Medicare	1148 (25.2)
Medicaid	215 (4.7)
Other or unknown	688 (15.1)
Socioeconomic status, median (IQR)^c^	3 (2-4)

Abbreviations: 1L, first line; CPS, combined positive score; ECOG, Eastern Cooperative Oncology Group; HPV, human papillomavirus; ICI, immune checkpoint inhibition; PD-L1, programmed death ligand 1.

^a^ Categorical variables shown as No. (%). Continuous variables shown as median (IQR).

^b^ Race was collected when recorded in the electronic health record (EHR) as part of routine clinical care. Other includes a myriad of unspecified terms describing race in the EHR and cannot be further categorized.

^c^ Five-level indicator of neighborhood socioeconomic conditions (1 indicates lowest, 5 indicates highest).

### Survival in Overall Population

**Figure 1.**
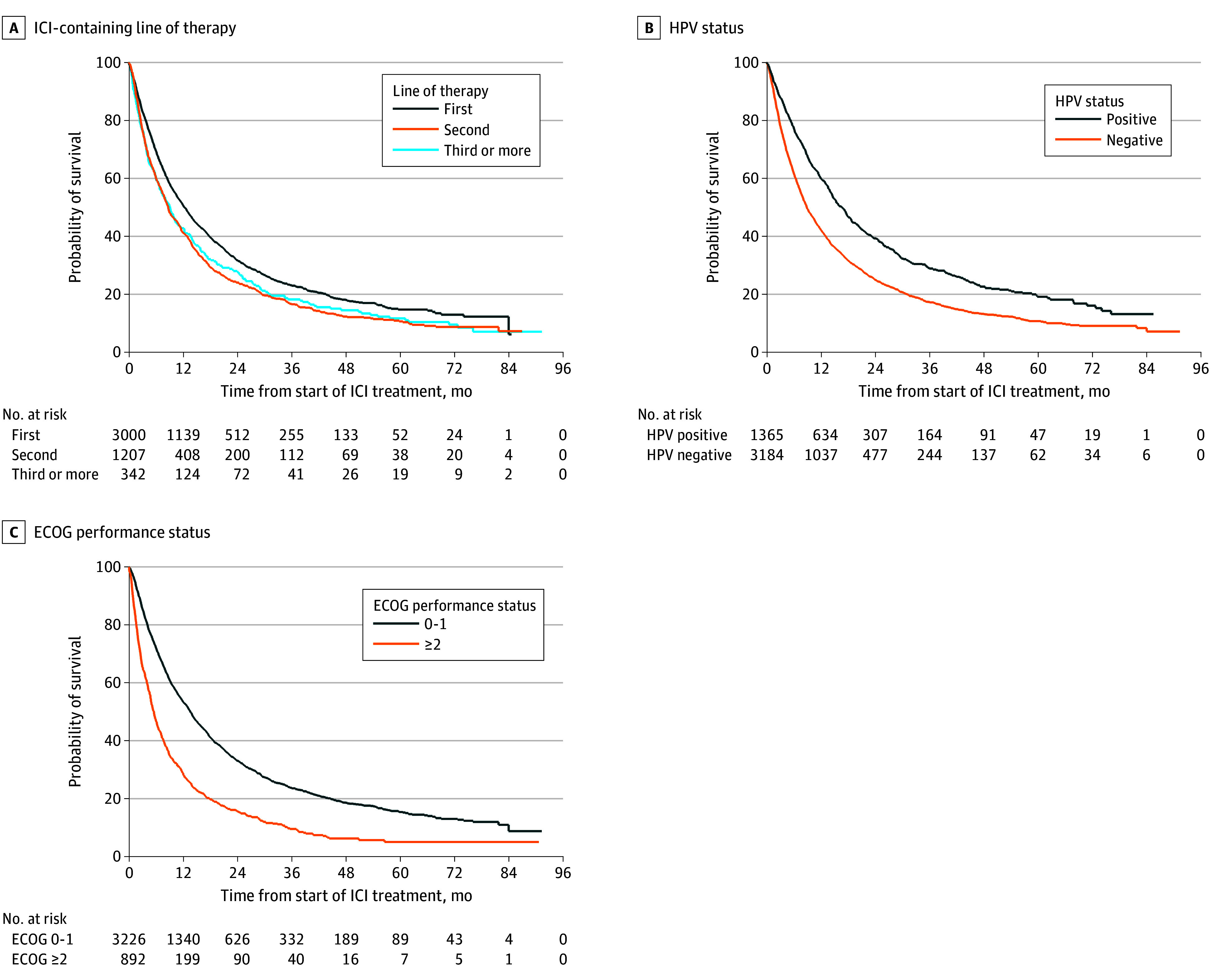
Kaplan-Meier Survival Estimates by Immune Checkpoint Inhibitor (ICI)-Containing Line of Therapy, Human Papillomavirus (HPV) Status, and Eastern Cooperative Oncology Group (ECOG) Performance Status

Median (IQR) overall survival (OS) from start of ICI-containing therapy was 10.9 (4.1-29.1) months in the total cohort (n = 4549). Median (IQR) OS was higher in patients who received ICI in frontline (12.2 [4.8-32.0] months) as compared with second-line therapy (8.7 [3.2-22.4] months) or third-line therapy (9.1 [3.3-25.8] months) (Figure 1A). Other factors associated with longer median (IQR) OS were HPV-positive oropharynx (16.6 [6.5-43.9] vs 8.8 [3.5-24.0] months) (Figure 1B) and ECOG performance status of 0 or 1 (13.5 [5.2-33.9] vs 5.5 [2.0-13.7] months) (Figure 1C). On adjusted multivariable regression, ICI in later LoT, non–HPV-positive oropharynx primary, and higher ECOG performance status remained associated with worse survival (eTable 1 in Supplement 1). No violation of proportional hazards was detected using Schoenfeld residual testing.

### Analysis of Patients Who Discontinued ICI at 1 and 2 Years

**Figure 2.**
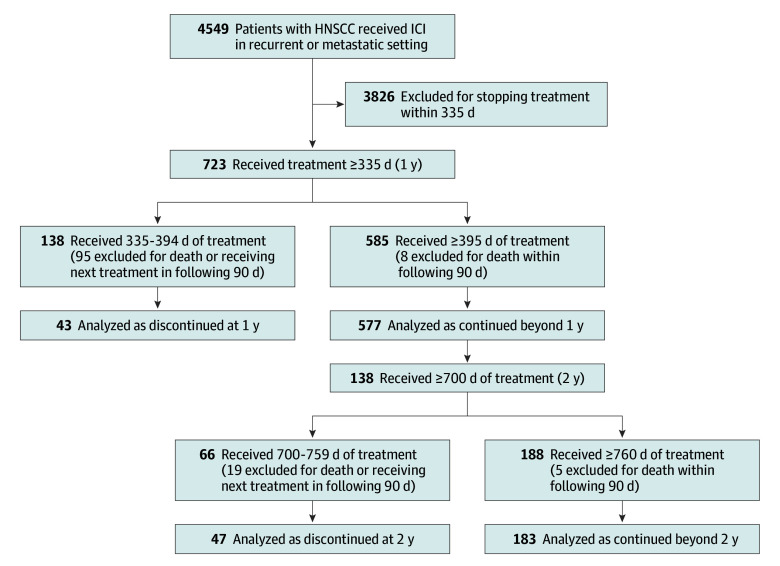
Discontinuation Analysis Diagram Diagram for inclusion and exclusion of patients for discontinuation analysis. HNSCC indicates head and neck squamous cell carcinoma; ICI, immune checkpoint inhibitor.

We identified 43 patients who stopped ICI at 1 year in the absence of next treatment or death, compared with 577 who continued treatment beyond 1 year; and 47 patients who stopped ICI at 2 years in the absence of next treatment or death, compared with 183 who continued beyond 2 years (Figure 2). Baseline characteristics were generally balanced between patients who stopped vs continued ICI at the 1- and 2-year time points, with the exception that stopping ICI at 2 years was more common at academic centers (eTable 2 in Supplement 1). Based on the sample sizes of the groups, log-rank test had 80% power to detect an HR of 0.55 (for the 1-year mark) and 0.54 (for the 2-year mark) at a 2-sided *P* value of .05.

**Figure 3.**
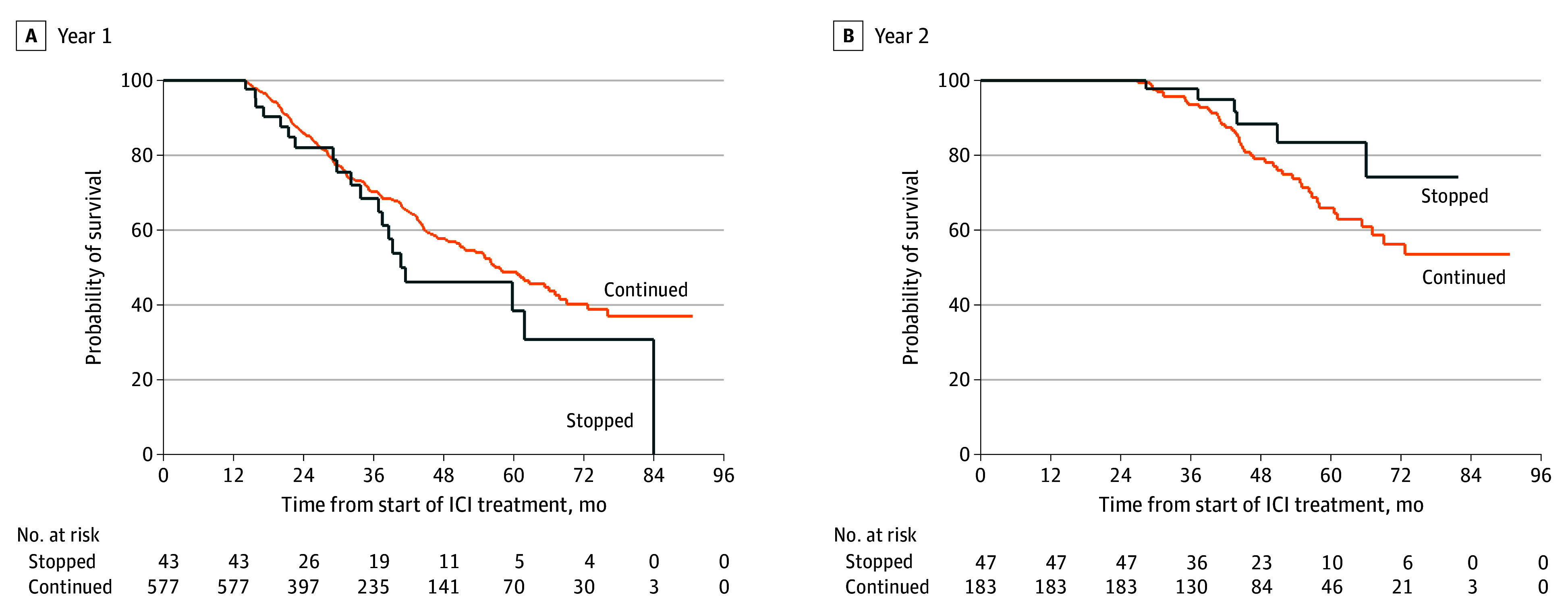
Kaplan-Meier Curve for Immune Checkpoint Inhibitor (ICI) Discontinuation vs Continuation at 1 Year and 2 Years

There was no statistically significant survival difference seen between patients who stopped and continued ICI at 1 and 2 years. Median OS for patients who discontinued vs continued at 1 year was 40.7 vs 57.7 months (HR, 1.33 [95% CI, 0.84-2.11]; *P* = .22) (Figure 3A); on multivariable regression, HR for death associated with discontinuation at 1 year was 1.26 (95% CI, 0.78-2.05; *P* = .35) (eTable 3 in Supplement 1). Median OS for patients who discontinued vs continued at 2 years was not reached (NR) vs NR (HR, 0.52 [95% CI, 0.22-1.22]; *P* = .13); on multivariable regression, HR for death associated with discontinuation at 2 years was 0.57 (95% CI, 0.21-1.52) (*P* = .26).

### Analysis of Patients Rechallenged With ICI

We identified 169 patients who were rechallenged with ICI without intervening therapy after at least a 60-day gap after stopping their initial ICI-containing LoT (immediate rechallenge), and 165 who were rechallenged with ICI after an intervening non-ICI therapy (most commonly carboplatin/paclitaxel or cetuximab) (delayed rechallenge).

**Figure 4.**
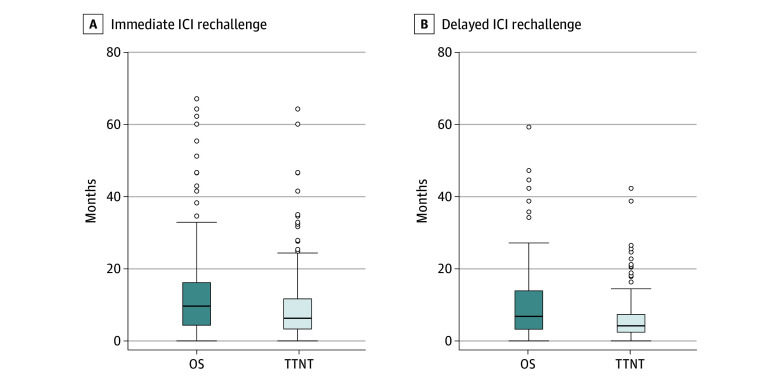
Overall Survival (OS) and Time to Next Treatment (TTNT) for Patients Undergoing Immediate Immune Checkpoint Inhibitor (ICI) Rechallenge or Delayed ICI Rechallenge Box-and-whisker plot of OS and TTNT for patients undergoing ICI rechallenge (A) and delayed ICI rechallenge (B). Box represents IQR (25th-75th percentile), with center line representing median value. The bottom and top whiskers are equal to the 25th percentile minus 1.5 times the IQR, and 75th percentile plus 1.5 times the IQR, respectively.

For the immediate rechallenge group (n = 169), ICI rechallenge occurred a median (IQR) of 6.4 (4.6-11.2) months after stopping the prior ICI-containing treatment; most received the same ICI agent (108 received pembrolizumab first, then pembrolizumab; 33 received nivolumab first, then nivolumab) whereas 12 received nivolumab after pembrolizumab and 15 received pembrolizumab after nivolumab. Only 16 patients were rechallenged with chemoimmunotherapy after at least a 60-day break after stopping ICI monotherapy; most patients were rechallenged with ICI monotherapy. Median OS from ICI rechallenge was 15.7 (95% CI, 13.7-21.9) months in the immediate rechallenge group, with 75 deaths among 169 patients. Median TTNT from immediate ICI rechallenge was 10.4 months (Figure 4A).

For the delayed rechallenge group (n = 165), ICI rechallenge occurred a median (IQR) of 5.8 (3.2-9.5) months after stopping ICI, suggesting a short interval of intervening chemotherapy. Median (IQR) OS from ICI delayed rechallenge was 9.9 (3.7-18.1) months, with 116 deaths among 165 patients. Median TTNT from delayed ICI rechallenge was 4.6 (2.6-9.0) months (Figure 4B).

## Discussion

To our knowledge, this study presents the largest observational cohort to date of over 4000 patients with R/M HNSCC treated with ICI-based therapy. Overall survival in our cohort closely mirrors clinical trial results: median OS in the frontline setting of 12.2 months was similar to the 12- to 13-month median OS observed in the KN-048 study^1^; and median OS of ICI in the second and third line of approximately 9 months in our cohort was slightly higher than the 7- to 8-month median OS seen in CM141^4^ and KN040.^3^ Notably, survival estimates for patients receiving second and later line ICI do not take into account time receiving earlier LoTs. Although median OS was shorter in the later line setting as compared with firstline ICI, our data suggest that ICI in later line settings still has utility if non–ICI-based frontline therapy was used. Unsurprisingly, HPV-negative cancers and patients with worse performance status had inferior survival.

Optimal duration of ICI treatment in long-term responders is an unanswered question in many malignant neoplasms including HNSCC, where pivotal trials administered ICI for up to 2 years. Our analysis shows that in clinical practice, discontinuation of ICI at 2 years is uncommon, with only approximately 1 in 5 patients stopping ICI in the absence of next treatment or death within 3 months, and even lower at community centers (a strikingly similar finding to our previous report in lung cancer).^6^

Although our analysis is limited by small numbers and possible residual confounding, we also show that there is no survival detriment on unadjusted or adjusted analysis associated with discontinuation of ICI at 2 years or even 1 year. The HR for death of less than 1 associated with discontinuation at 2 years may indicate that the 2-year discontinuation cohort represents a highly selected, favorable-prognosis population deemed appropriate for surveillance. Alternatively, treatment discontinuations may reflect occurrence of immune-related adverse events (IRAEs), which have been associated with ICI benefit and longer survival in HNSCC.^11,12^ Prior reports have suggested that deep durable responses can be sustained for many years after treatment termination in HNSCC^13^; retrospective studies in other ICI-treated malignant neoplasms have suggested that depth of response may correlate with survival after ICI discontinuation in long-term responders,^14,15^ and that indefinite duration ICI does not lead to prolonged survival compared with 2-year fixed duration treatment.^6^ Similar to these reports, our data do not suggest benefit to continuing ICI indefinitely. Stopping ICI treatment at 2 years may be a reasonable strategy that does not compromise survival and may reduce the risk of long-term medical toxic effects and high financial costs.

We also present an analysis of ICI rechallenge after either at least 60 days off all systemic therapy (immediate rechallenge), which may represent resumption after treatment pause for toxic effects, or intervening non-ICI chemotherapy (delayed rechallenge), presumably in the setting of disease progression. In both cohorts, median OS after ICI rechallenge was promising (15.7 and 9.9 months, respectively). Median TTNT of 10.4 and 4.6 months, respectively, suggests that these patients are deriving benefit from ICI rechallenge. It must be noted that the immediate rechallenge group had a relatively long treatment-free interval and likely represent a highly selected group that did not need urgent intervening chemotherapy; thus, the survival and TTNT estimates should not be interpreted as a comparison of efficacy between immediate and delayed ICI rechallenge.

### Limitations

This study has limitations. A major limitation of this analysis is that the reasons for ICI discontinuation and resumption (eg, progression, toxicity, patient preference) are not known. Rechallenge may have occurred in the setting of disease progression or recovery from IRAE and is not without risk; a pharmacovigilance study found that in patients who discontinued ICI due to IRAE, rechallenge was associated with recurrence of the same IRAE in 28.8% of cases.^16^ Nevertheless, this exploratory analysis indicates that ICI rechallenge can yield meaningful efficacy in a subset of patients. Other important data elements not available for these analyses include response status as defined by Response Evaluation Criteria in Solid Tumors, dates of progression, detailed toxicity information, type of HPV testing, and information on palliative locally ablative therapy including radiation. Additionally, there were some missing data in variables including PD-L1 status, ECOG performance status, and pattern of recurrence. Additionally, as this study was conducted using a United States–based observational database, findings are not completely generalizable to a global population.

## Conclusions

Despite limitations inherent in any retrospective analysis, we believe that this large observational cohort study of ICI treatment in R/M HNSCC reveals several important findings with management implications. First, survival with ICI-based therapy in US oncology practices closely mirrors survival in clinical trials both in the frontline and second- and later-line settings; patients who did not receive ICI as a frontline treatment, as well as those who have ECOG performance status of at least 2, may still derive benefit from ICI therapy. Second, indefinite duration ICI treatment in HNSCC does not appear to provide survival benefit compared with limited 1- or 2-year duration treatment. Finally, ICI rechallenge can provide meaningful clinical benefit in some patients. Further study is needed to validate factors beyond PD-L1 CPS score and disease burden (eg, circulating tumor DNA, molecular profiling) to tailor treatment and management strategies for this disease.

## Notes

Supplement 1. **eTable 1.** Overall Survival Multivariable Regression, Full Cohort **eTable 2.** Baseline Characteristics, Continuation vs Discontinuation Cohorts at 1 and 2 Years **eTable 3.** Multivariable Regression/HR of Death Associated With Treatment Discontinuation **eFigure.** Definition of ICI Continuation/Discontinuation at 1 and 2 Years

Supplement 2.
Data Sharing Statement
