# Supplementary material for: Overall Survival, Treatment Duration, and Rechallenge Outcomes With ICI Therapy for Recurrent or Metastatic HNSCC
Source: JAMA Netw Open. 2024 Aug 19;7(8):e2428526. doi: 10.1001/jamanetworkopen.2024.28526 (PMC11333980; doi:10.1001/jamanetworkopen.2024.28526)
Supplement: Supplement 1. — eTable 1. Overall Survival Multivariable Regression, Full Cohort eTable 2. Baseline Characteristics, Continuation vs Discontinuation Cohorts at 1 and 2 Years eTable 3. Multivariable Regression/HR of Death Associated With Treatment Discontinuation eFigure. Definition of ICI Continuation/Discontinuation at 1 and 2 Years [file jamanetwopen-e2428526-s001.pdf]

Supplemental Online Content

Sun L, Cohen RB, D'Avella CA, Singh AP, Schoenfeld JD, Hanna GJ. Overall survival, treatment duration, and immunotherapy rechallenge outcomes among patients receiving immune checkpoint inhibitor therapy for recurrent or metastatic head and neck squamous cell carcinoma. *JAMA Netw Open.* 2024;7(8):e2428526. doi:10.1001/jamanetworkopen.2024.28526

eTable 1. Overall Survival Multivariable Regression, Full Cohort

eTable 2. Baseline Characteristics, Continuation vs Discontinuation Cohorts at 1 and 2 Years

eTable 3. Multivariable Regression/HR of Death Associated With Treatment Discontinuation

eFigure. Definition of ICI Continuation/Discontinuation at 1 and 2 Years

This supplemental material has been provided by the authors to give readers additional information about their work.

© 2024 Sun L et al. *JAMA Network Open*.

eTable 1. Overall survival multivariable regression, full cohort

Characteristic	HR for death	95% confidence interval	p-value
ICI in 2nd line treatment (ref: 1st line)	1.13	1.06-1.19	<0.01
Age	1.00	1.00-1.00	0.86
Male	1.07	0.98-1.17	0.14
Race (ref: white)			
Black/African American	0.93	0.80-1.09	0.39
Asian	0.99	0.72-1.37	0.96
Other/Unknown	1.04	0.94-1.14	0.46
History of Smoking	1.02	0.93-1.12	0.67
Primary site (ref: HPV+ OP)			
HPV- Oropharynx	1.40	1.25-1.56	<0.01
Larynx	1.27	1.14-1.42	<0.01
Oral Cavity	1.77	1.59-1.97	<0.01
Hypopharynx	1.37	1.17-1.61	<0.01
Unknown primary	0.85	0.66-1.09	0.19
ECOG performance status (ref: 0)			
1	1.24	1.13-1.36	<0.01
2	1.98	1.77-2.22	<0.01
3	2.98	2.45-3.63	<0.01
4	1.76	0.87-3.58	0.12
Unknown	1.31	1.13-1.51	<0.01
Year of treatment	0.99	0.97-1.02	0.53
PD-L1 CPS (ref:0)			
1-19	0.99	0.85-1.15	0.88
20	0.88	0.76-1.03	0.12
Unknown	1.10	0.96-1.26	0.15
Socioeconomic status (ref: 1)[a]			
2	1.01	0.90-1.14	0.83
3	0.95	0.84-1.07	0.38
4	0.91	0.81-1.03	0.13
5	0.87	0.76-1.00	0.04
Unknown	1.05	0.90-1.21	0.56
Academic practice	0.92	0.84-1.00	0.06
Insurance (ref:private)			
Medicare	0.96	0.88-1.05	0.41
Medicaid	0.84	0.71-1.00	0.05
Other or unknown	0.94	0.85-1.05	0.30

[a] 5-level indicator of neighborhood socioeconomic conditions (1 - lowest SES; 5 - highest SES).
HR, hazard ratio; ICI, immune checkpoint inhibitor; AA, African American; HPV, human papillomavirus; ECOG, Eastern Cooperative Oncology Group; CPS, combined positive score;

© 2024 Sun L et al. *JAMA Network Open*.

eTable 2. Baseline characteristics, continuation vs discontinuation cohorts at 1 and 2 years

		Continued beyond 1 year	Stopped at 1 year	p-value	Continued beyond 2 years	Stopped at 2 years	p-value
		N=577	N=43		N=183	N=47	
Age		65 (59-71)	65 (55-72)	0.84	65 (57-71)	67 (58-73)	0.3
Male		461 (79.9%)	31 (72.1%)	0.22	145 (79.2%)	37 (78.7%)	0.94
Race	White	385 (66.7%)	28 (65.1%)	0.41	115 (62.8%)	37 (78.7%)	0.44
	Black/AA	28 (4.9%)	4 (9.3%)		12 (6.6%)	2 (4.2%)	
	Asian	4 (0.7%)	1 (2.3%)		2 (1.1%)	0 (0.0%)	
	Other/Unknown	160 (27.7%)	10 (23.4%)		54 (29.5%)	8 (17.0%)	
Year of 1L treatment	2015-2017	129 (22.4%)	11 (25.6%)	0.85	56 (30.6%)	9 (19.1%)	0.1
	2018-2020	268 (46.4%)	20 (46.5%)		101 (55.2%)	34 (72.3%)	
	2021-2023	180 (31.2%)	12 (27.9%)		26 (14.2%)	4 (8.5%)	
ECOG PS	0	177 (30.7%)	11 (25.6%)	0.72	58 (31.7%)	15 (31.9%)	0.94
	1	285 (49.4%)	21 (48.8%)		82 (44.8%)	23 (48.9%)	
	2	59 (10.2%)	7 (16.3%)		18 (9.8%)	4 (8.5%)	
	3	5 (0.9%)	1 (2.3%)		2 (1.1%)	0 (0.0%)	
	4	2 (0.3%)	0 (0.0%)		2 (1.1%)	0 (0.0%)	
	Unknown	49 (8.5%)	3 (7.0%)		21 (11.5%)	5 (10.6%)	
Smoking history		450 (78.0%)	33 (76.7%)	0.85	146 (79.8%)	37 (78.7%)	0.87
Primary site	HPV+ Oropharynx	216 (37.4%)	16 (37.2%)	0.53	70 (38.3%)	19 (40.4%)	0.54
	HPV- Oropharynx	93 (16.1%)	5 (11.6%)		35 (19.1%)	5 (10.6%)	
	Larynx	125 (21.7%)	12 (27.9%)		36 (19.7%)	7 (14.9%)	
	Oral Cavity	86 (14.9%)	8 (18.6%)		24 (13.1%)	10 (21.3%)	
	Hypopharynx	34 (5.9%)	0 (0.0%)		9 (4.9%)	3 (6.4%)	
	Unknown primary	23 (4.0%)	2 (4.7%)		9 (4.9%)	3 (6.4%)	
PD-L1 CPS	0	43 (7.5%)	4 (9.3%)	0.44	14 (7.7%)	3 (6.4%)	0.9
	1-19	94 (16.3%)	11 (25.6%)		23 (12.6%)	8 (17.0%)	
	≥20	105 (18.2%)	8 (18.6%)		26 (14.2%)	8 (17.0%)	
	Unknown	304 (52.7%)	20 (46.5%)		115 (62.8%)	27 (57.4%)	
Line of 1st ICI-containing therapy	1	402 (69.7%)	28 (65.1%)	0.77	125 (68.3%)	32 (68.1%)	0.036
	2	135 (23.4%)	11 (25.6%)		39 (21.3%)	15 (31.9%)	
	3+	40 (6.9%)	4 (9.3%)		19 (10.4%)	0 (0.0%)	
1st ICI containing line of therapy	ICI monotherapy	452 (78.3%)	37 (86.0%)	0.23	147 (80.3%)	41 (87.2%)	0.27
	ICI+chemo	125 (21.7%)	6 (14.0%)		36 (19.7%)	6 (12.8%)	
Practice Type	Community	456 (79.0%)	34 (79.1%)	1	153 (83.6%)	32 (68.1%)	0.017
	Academic	121 (21.0%)	9 (20.9%)		30 (16.4%)	15 (31.9%)	
Geographic Location	West	58 (13.2%)	4 (12.5%)	0.52	22 (14.9%)	5 (16.1%)	0.99
	Midwest	62 (14.1%)	2 (6.3%)		18 (12.2%)	4 (12.9%)	
	Northeast	40 (9.1%)	2 (6.3%)		13 (8.8%)	3 (9.7%)	
	South	279 (63.6%)	24 (75.0%)		95 (64.2%)	19 (61.3%)	
Insurance type	Commercial	314 (54.4%)	23 (53.5%)	0.98	101 (55.2%)	30 (63.8%)	0.39
	Medicare	139 (24.1%)	10 (23.3%)		38 (20.8%)	11 (23.4%)	

© 2024 Sun L et al. *JAMA Network Open*.

	Medicaid	31 (5.4%)	2 (4.7%)		12 (6.6%)	1 (2.1%)	
	Other or unknown	93 (16.1%)	8 (18.6%)		32 (17.5%)	5 (10.6%)	
SES[a]		3 (2-4)	3 (2-5)	0.61	3 (2-4)	3 (2-4)	0.7

[a] 5-level indicator of neighborhood socioeconomic conditions (1 - lowest SES; 5 - highest SES). IQR, interquartile range; 1L, first line; ECOG, Eastern Cooperative Oncology Group; HPV, human papillomavirus; CPS, combined positive score; ICI, immune checkpoint inhibition; PD1, programmed death ligand 1

Supplemental Table 3. Multivariable regression/HR of death associated with treatment discontinuation

	DISCONTINUATION AT 1 YEAR			DISCONTINUATION AT 2 YEARS		
	HR for death	95% confidence interval	p-value	HR for death	95% confidence interval	p-value
**Discontinuation (ref: continuation)**	**1.26**	**0.78-2.05**	**0.349**	**0.57**	**0.21-1.52**	**0.261**
Age	1.02	1.01-1.04	0.004*	1.05	1.01-1.09	0.013*
Male	1.04	0.72-1.49	0.834	2.42	0.81-7.25	0.114
Race (ref: white)						
Black/African American	0.90	0.48-1.72	0.760	0.86	0.23-3.23	0.820
Asian	1.40	0.40-4.93	0.599	-	- -	-
Other	1.00	0.68-1.46	0.983	1.33	0.50-3.55	0.572
Unknown	0.62	0.37-1.05	0.077	0.50	0.15-1.69	0.265
History of Smoking	1.10	0.74-1.64	0.633	1.26	0.45-3.48	0.660
Primary site (ref: HPV+ OP)						
HPV- Oropharynx	1.08	0.69-1.69	0.747	1.31	0.46-3.76	0.614
Larynx	1.66	1.14-2.42	0.008*	2.22	0.85-5.81	0.103
Oral Cavity	1.10	0.67-1.79	0.706	2.00	0.58-6.87	0.270
Hypopharynx	2.19	1.23-3.90	0.008*	8.65	2.53-29.57	0.001*
Unknown primary	0.35	0.13-0.97	0.043*	0.29	0.03-2.79	0.283
ECOG performance status (ref: 0)						
1	1.34	0.95-1.90	0.093	1.11	0.51-2.42	0.784
2	1.79	1.12-2.86	0.015*	1.72	0.56-5.30	0.344
3	4.92	1.65-14.67	0.004*	67.42	6.78-670.83	0.000*
4	1.56	0.19-12.90	0.681	6.47	0.53-78.94	0.143
Unknown	0.83	0.44-1.56	0.560	1.24	0.39-3.95	0.714
Year of treatment	0.96	0.86-1.08	0.525	1.08	0.76-1.54	0.654
PD-L1 CPS (ref:0)						
1-19	0.72	0.37-1.38	0.317	0.42	0.09-2.05	0.285
20	0.76	0.40-1.44	0.401	0.14	0.01-1.35	0.089
Unknown	0.64	0.37-1.12	0.120	0.66	0.20-2.25	0.511
Socioeconomic status (ref: 1)[a]						
2	0.75	0.47-1.20	0.230	0.41	0.13-1.26	0.120
3	0.71	0.46-1.09	0.121	0.54	0.21-1.36	0.192
4	0.69	0.45-1.08	0.102	0.24	0.08-0.69	0.008*
5	0.69	0.41-1.14	0.147	0.44	0.14-1.36	0.152
Unknown	0.83	0.43-1.62	0.595	0.75	0.21-2.68	0.659
Academic practice	0.71	0.48-1.04	0.077	1.04	0.38-2.86	0.934
Insurance (ref:private)						
Medicare	1.08	0.77-1.52	0.650	0.52	0.22-1.22	0.134
Medicaid	1.73	0.97-3.08	0.062	1.51	0.38-5.99	0.558
Other or unknown	1.18	0.79-1.77	0.414	0.95	0.41-2.21	0.902
Line of ICI-containing treatment	1.02	0.82-1.28	0.836	0.82	0.48-1.40	0.461

[a] 5-level indicator of neighborhood socioeconomic conditions (1 - lowest SES; 5 - highest SES).

© 2024 Sun L et al. *JAMA Network Open*.

HR, hazard ratio; ICI, immune checkpoint inhibitor; AA, African American; HPV, human papillomavirus; ECOG, Eastern Cooperative Oncology Group; CPS, combined positive score.

© 2024 Sun L et al. *JAMA Network Open*.

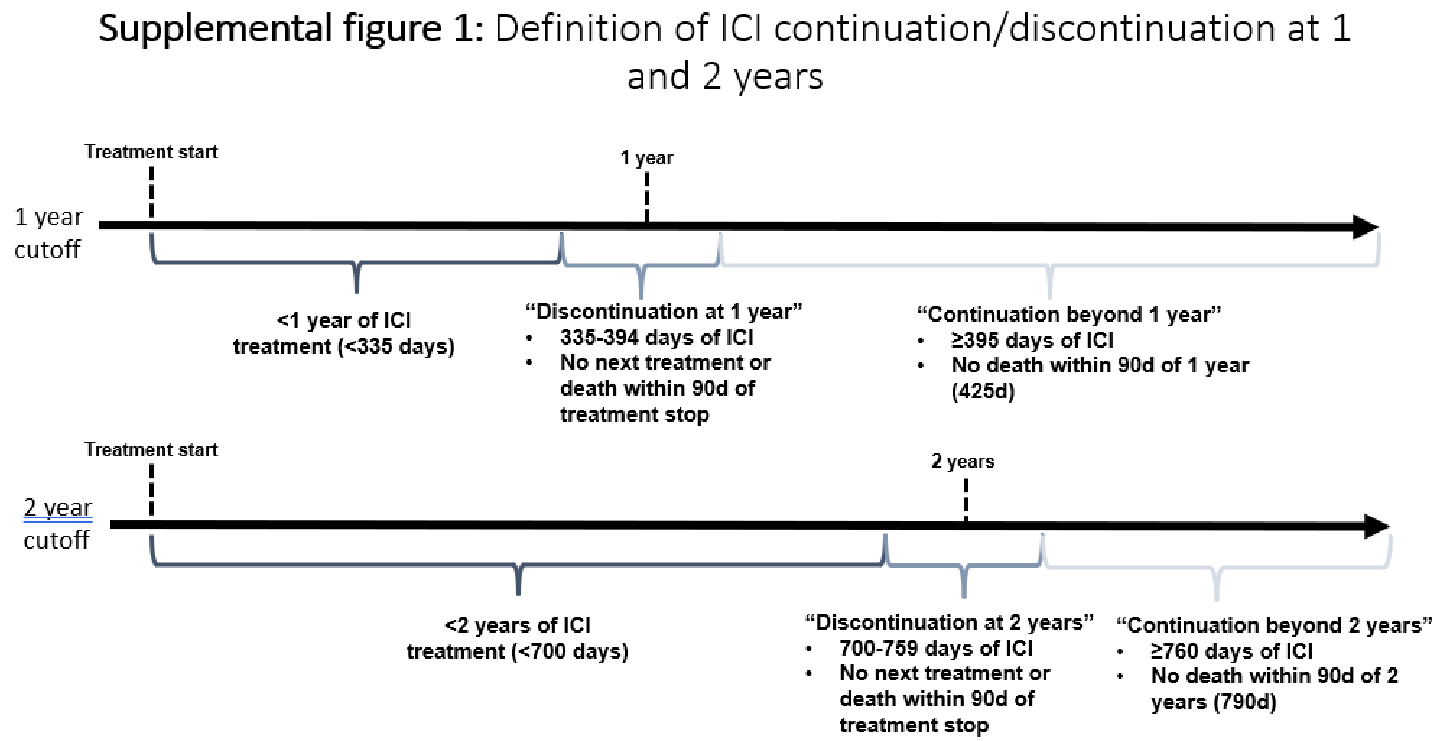

eFigure. Definition of ICI Continuation/Discontinuation at 1 and 2 Years

© 2024 Sun L et al. *JAMA Network Open*.
