# Supplementary material for: Overall Survival, Treatment Duration, and Rechallenge Outcomes With ICI Therapy for Recurrent or Metastatic HNSCC
Source: JAMA Netw Open. 2024 Aug 19;7(8):e2428526. doi: 10.1001/jamanetworkopen.2024.28526 (PMC11333980; doi:10.1001/jamanetworkopen.2024.28526)
Supplement: Supplement 2. — Data Sharing Statement [file jamanetwopen-e2428526-s002.pdf]

Data Sharing Statement

Sun. Overall Survival, Treatment Duration, and Immunotherapy Rechallenge Outcomes Among Patients Receiving Immune Checkpoint Inhibitor Therapy for Recurrent or Metastatic Head and Neck Squamous Cell Carcinoma. *JAMA Netw Open*. Published August 16, 2024. doi:10.1001/jamanetworkopen.2024.28526

Data
Data available: No
